# Evaluating the Current Evidence for the Efficacy of Cannabis in Symptom Management of Endometriosis-Associated Pain

**DOI:** 10.1089/imr.2024.0017

**Published:** 2024-08-19

**Authors:** Savannah Calleson Cummings, Nicole Ennis, Katie Kloss, Robyn Rosasco

**Affiliations:** College of Medicine, FL State University, Tallahassee, Florida, USA

**Keywords:** endometriosis, inflammation, cannabis, pain management, women’s health

## Abstract

**Introduction:**

Endometriosis is a chronic condition that affects millions of women in the United States. Chronic pain can be debilitating and complex to manage. Despite common approaches like hormonal treatments and surgery, many women continue to endure pain. Managing the chronic and multifaceted nature of endometriotic pain necessitates a comprehensive strategy.

**Methods:**

This study explores the potential of cannabis in alleviating endometriosis-related pain through an extensive literature search. Cohort studies, case-control studies, cross-sectional surveys, and systematic reviews from January 1996 to August 2022 were eligible for inclusion. Articles were then assessed and included for further analysis if they met the following criteria: (1) Population was women with endometriosis; (2) Discussed the therapeutic use of medical cannabis for endometriosis-related pain.

**Results:**

The literature search revealed 522 articles, with eight articles meeting criteria for analysis: four cross-sectional surveys, two systematic reviews, one retrospective cohort study, and one literature review. Cannabis consumption for symptom relief was common among women with endometriosis, and some even reported that the use of cannabis reduced their use of analgesics.

**Discussion:**

The use of cannabis for endometriosis-related pain holds significant promise for addressing the often-debilitating comfort experienced by those with this condition. By interacting with the endocannabinoid system, cannabis may provide relief by modulating pain perception, reducing inflammation, relaxing muscles, and alleviating neuropathic discomfort.

## Background

Endometriosis is a common, chronic inflammatory gynecological condition that affects approximately 6.5 million women in the US and 190 million women worldwide.^1^ It is characterized by the presence of endometrial-like tissue found outside the uterus. Endometriosis causes a myriad of symptoms such as chronic pelvic pain, dyspnea, dyspareunia, dyschezia, and dysuria. Management of the chronic and life-limiting health implications of endometriosis has been associated with decreased quality of life among women coping with this disease.^2^ Typical management of endometriosis often includes prescription and nonprescription analgesics, oral contraceptives, laparoscopy, and in some cases hysterectomy.^3^ Despite its prevalence, there is no cure for this chronic illness. Furthermore, traditional treatment options are limited and have come with adverse effects, which has resulted in a growing interest in alternative approaches for effective pain management. In recent years within the United States, there has been a surge in cannabis legalization for various medical conditions, including chronic pain management.^4^ Specifically, medical cannabis refers to the use of the marijuana plant and its derivatives for therapeutic purposes under the guidance of a medical professional. Growing access to medical marijuana, along with the analgesic and anti-inflammatory properties of cannabinoids, has sparked interest in the role of cannabinoids in alleviating endometriosis-related pain.

However, the relationship between medical cannabis and endometriosis-related pain remains unclear. A comprehensive analysis and evaluation of the current evidence is necessary to determine the next steps needed to advance scientific knowledge regarding the relationship between cannabis use and management of endometriosis-associated pain. The current perspective presents our review of the literature on cannabis and the management of endometrial-associated pain and outlines the next steps in this line of research, with a model for how and why cannabis should be examined as a first-line treatment for the management of endometriosis-related pain.

## Methods

Prior to starting our review, this protocol was registered with PROSPERO (Registration # CRD42022353816). The literature for this review was collected from the following databases: PubMed, Web of Science, Cochrane Central Register of Controlled Trials, MEDLINE, and Embase. We included the following terms in our electronic search strings: MeSH and other database-specific subject headings, synonym keyword terms and phrases, and a partially validated search filters^5–7^ The search was designed by a health sciences librarian, and peer review of the search was conducted by a second health sciences librarian using the Peer Review of Electronic Search Strategies checklist and form. This search included only human subject research studies. We excluded animal research studies from the searches using a partially validated search filter when appropriate for the database. We also restricted our searches to only include English-language studies published after January 1, 1996. Searches were run in each database and exported into an EndNote library. All search results were then uploaded into Covidence and deduplicated upon importation. We used Covidence to organize, screen, review, and extract data from the unique references retrieved in the searches.

Cohort studies, case-control studies, cross-sectional surveys, and systematic reviews from January 1996 to August 2022 assessing any relationship between cannabis use and endometriosis were eligible for inclusion. Retrieved articles were independently reviewed by two reviewers and any discrepancies were resolved by consensus with a third reviewer. Suitable articles were assessed to see if they met the following criteria: (1) Population was women with endometriosis; (2) Discussed the therapeutic use of medical cannabis for endometriosis-related pain.

## Results

A total of 522 references were identified and retrieved. Following screening, eight articles were included for review: four cross-sectional surveys, two systematic reviews, one retrospective cohort study, and one review of literature (Table 1). Across five of the studies, 1817 total participants were found to have used cannabis for endometriosis symptom management. All included studies were from countries where cannabis is legalized medicinally and/or recreationally.

**Table 1.** Characteristics of Included Studies

Authors	Title	Country of origin	Aims/purpose	Research design	Target population
Armour, M., Sinclair, J., Chalmers, J., & Smith, C.	Self-Management Strategies amongst Australian Women with Endometriosis: A National Online Survey	Australia	The aim of this survey was to determine the prevalence of use, safety, and self-rated effectiveness of common forms of self-management.	Cross-sectional study	Women aged 18–45 living in Australia with a dx of endometriosis recruited via social media using endometriosis support & advocacy groups
Armour, M., Sinclair, J., Chalmers, J., & Smith, C.	Endometriosis and cannabis consumption during the covid-19 pandemic: an international cross-sectional survey	Australia	As cannabis is a relatively common form of self-management in endometriosis, this study aims to explore the impact of the COVID-19 pandemic on cannabis consumption in those with endometriosis.	Cross-sectional study	People from various countries aged 18–55 who had been told by their medical doctor that they had endometriosis
Armour, M., Sinclair, J., Noller, G., & Girling, J.	Illicit Cannabis Use as a Management Strategy in New Zealand Women with Endometriosis: an online survey	Australia	The aim of this survey was to explore New Zealanders’ use of cannabis where endometriosis was self-identified as a condition that was being treated with cannabis, as well as the impact of cannabis use on the usage on other pharmaceuticals.	Cross-sectional study	Convenience sample of people in New Zealand self-reporting therapeutic use of cannabis or a cannabis-based medicine across multiple self-identified conditions
Liang, A., Gingher, E. L., & Coleman, J. S.	Medical Cannabis for Gynecologic Pain Conditions: A Systematic Review	United States	This study sought to characterize patterns of cannabis use for gynecologic pain and its effectiveness as an analgesic.	Systematic Review	Non-pregnant adult women who used cannabinoids for gynecologic pain conditions (e.g., chronic pelvic pain, vulvodynia, endometriosis, interstitial cystitis, m alignancy)
Marcu, I., Gee, A., & Lynn, B.	Cannabinoids and Chronic Pelvic Pain in Women: Focus on Endometriosis	United States	This review focuses on the interaction of the endocannabinoid system with the menstrual cycles, with endometriotic lesions, and within the bladder, and provides an overview of existing literature of the effects of endocannabinoids on chronic pain generally, with a focus on neuropathic pain.	Review	The search parameters for this review were not described.
Sinclair, J., Collett, L., Abbott, J., Pete, D.W., & Sarris, J.	Effects of Cannabis Ingestion on Endometriosis Associated pelvic pain and related symptoms	Australia	The goal of this study was to investigate the self-rated effectiveness, dosage forms, dosage amount, and cannabinoid ratios of quality-assured legal cannabis products that women are using in the Canadian regulated market, using the Strainprint smartphone application, which tracks the legal cannabis product allows indivduals to track medicinal cannabis use.	Cohort study	Participants who self-reported having endometriosis
Sinclair, J., Smith, C.A., Abbott, J., & Chalmers, K.J.	Cannabis use, a self- management strategy among Australian women with endometriosis: results from a national online survey	Australia	The aim of this study was to determine the prevalence of cannabis use, cost, tolerability, self-rated effectiveness, and possible changes in pharmaceutical use reported by Australian women with endometriosis.	Cross-sectional study	Women living in Australia with surgically confirmed endometriosis
Taylor, C., & Birch, B.	Cannabinoids in Urology. Which Benign Conditions Might They Be Appropriate to Treat: A Systematic Review	United Kingdom	This review aims to identify how cannabinoids are currently used in the treatment of bladder dysfunction, lower urinary tract symptoms, chronic pelvic pain syndrome/chronic prostatitis, interstitial cystitis/painful bladder syndrome, endometriosis, and in renal pathologies as well as in generic urological pain management.	Systematic Review	Patients with specific benign urological pathologies that have been exposed to a form of cannabinoid treatment or adjunct therapy

Self-reported cross-sectional survey data suggests that cannabis is beneficial for management of endometriosis-related pain. Cross-sectional survey data currently constitutes the bulk of the available scientific evidence in this domain to date and has largely been collected by the same researchers. Survey data of self-management strategies among women with endometriosis showed that cannabis use was reported with the greatest effectiveness for alleviation of symptoms.^8^ This data also suggest that use of cannabis for symptom relief is common, and countries with legal access were found to have greater use of cannabis for managing endometriosis-related symptoms than those without legal access.^9^ Additional self-reported survey data shows cannabis was efficacious in reducing pelvic pain, improving sleep, and reducing anxiety and depression, as well as managing symptoms of nausea and vomiting that can be associated with endometriosis.^8–11^ Data also showed an inclination for women to favor inhaled delivery of cannabis due to the rapid onset of effects and symptom alleviation compared with oral products.^12^

Overall, results showed that cannabis consumption for symptom relief was common among women with endometriosis, and some women even reported that use of cannabis reduced their use of analgesics. All retrieved articles noted the need for future studies investigating cannabis use for pelvic pain, as well as the lack of clinical human studies to confirm what we know from animal studies.

## Discussion

Endometriosis pain management is multifaceted and unique to each individual. The current evidence suggests potential therapeutic benefits of medical cannabis for endometriosis related pain. Most of the available data comes from self-reported cross-sectional surveys that have consistently shown that cannabis use is associated with the relief of endometriosis-associated symptoms compared with traditional therapies such as analgesics and heating pads.^13^ Our analysis identified a clear lack of randomized controlled trials on the use of cannabis for endometriosis-related pain, indicating an important direction for future research given the recent findings that dysfunction in the endocannabinoid system (ECS) may play a role in endometriosis-related pain.^14^

Marijuana, including cannabinoids such as THC and CBD that impact the endocannabinoid system, have the potential to effectively alleviate pain in several gynecological conditions, including chronic pelvic pain and endometriosis.^15,16^ Cannabinoid receptors are a part of a signaling system involved in many physiological processes, including pain sensation, appetite, and immune function. Expression of cannabinoid receptors (CB1 and CB2) has been shown in the endometrial glands of women with endometriosis, whereas no expression is seen in women with normal ovarian tissue.^14^ Endometriotic lesions found in the ovaries are driven by the cyclic hormonal changes of the body and are the most common source of pain during the menstrual cycle.^17^ Preclinical studies using animal models have shown promising results regarding antiproliferative effects of cannabinoids on endometriotic lesions, inhibiting the growth and spread of endometrial tissue outside the uterus in animal studies.^18^

Inflammation plays a significant role in the development and progression of endometriosis. Several studies have investigated the anti-inflammatory effects of cannabis and its potential impact on endometriosis-associated inflammation. Cannabinoids have been shown to possess immunomodulatory properties and can reduce the release of inflammatory mediators.^14,19^ Preclinical studies have demonstrated that cannabinoids can attenuate inflammatory responses in endometriosis models.^14,18–21^ These findings suggest that cannabis may have potential anti-inflammatory effects that could be beneficial in managing endometriosis-related inflammation and associated pain.

**FIG. 1.**
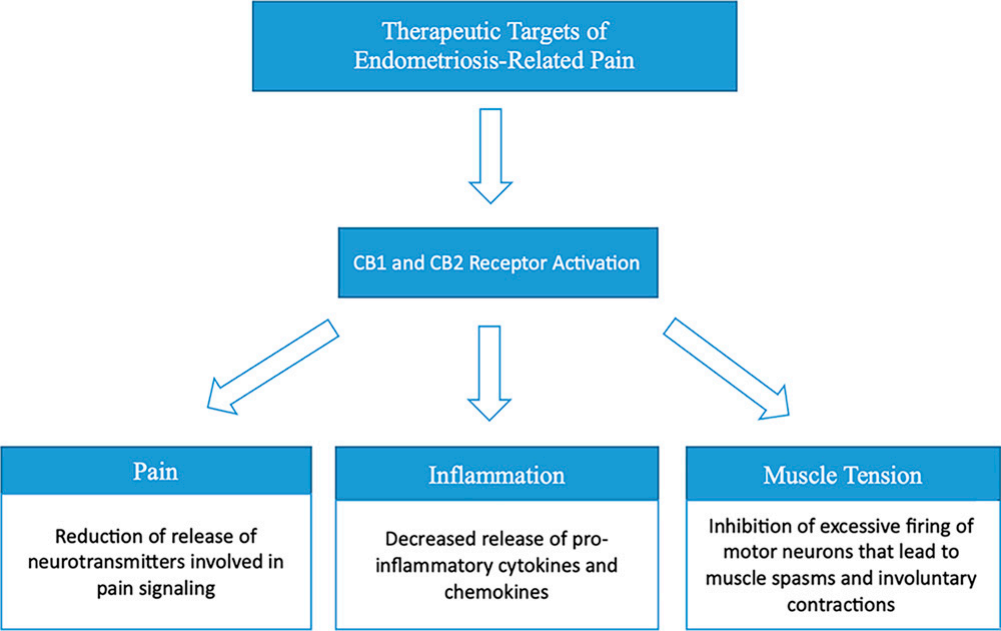
Therapeutic Targets of Cannabis for Relieving Endometriosis-Related Pain. This flow diagram illustrates the key therapeutic targets within the endocannabinoid system that interact with cannabis compounds. The interaction depicted above provide insights into the potential pathways for the applications of cannabis in the management of endometriosis-related pain.

Muscle cramps are a common symptom experienced by women with endometriosis, often causing severe, debilitating pain. Cannabinoids have antispasmodic effects, which can help relieve muscle tension and spasms identifying the potential for cannabis to reduce pain associated with endometriosis-related cramps (Fig. 1).

Management of endometriosis typically includes a combination of medical and surgical interventions. Specific management is subject to variation based on severity of symptoms and desire for fertility, as well as individual patient preferences and history. For severe cases, surgery is often recommended. As no surgery comes without risks, the main goal of management of endometriosis is to alleviate symptoms and prevent the growth of further inappropriately located endometrial tissue. Emerging evidence suggests the endocannabinoid system, which regulates various physiological processes including pain perception and inflammation, may play a role in the pathophysiology of endometriosis. The ECS consists of cannabinoid receptors (CB1 and CB2) and endogenous cannabinoids (endocannabinoids) that bind to these receptors.^18^ By targeting the ECS, cannabis may modulate pain signaling pathways and reduce pain intensity.^22^ Research suggests that cannabis may act a potential analgesic agent, providing relief to individuals with chronic pain, including those with endometriosis (Fig. 1).^23–24^

The cannabinoid receptor 1 (CB1) is found in the amygdala, hippocampus, and cerebral cortex, areas that are highly associated with anxiety.^25^ Anxiety is understood to be a complex biological process that can result from inappropriate activation of these receptors and circuits in the brain. As the endocannabinoid system plays a large role in modulation of these circuits, cannabinoids can have a biphasic effect on anxiety. As survey data suggest that women with endometriosis and/or polycystic ovarian syndrome commonly suffer from depression and/or anxiety in addition to their gynecological diagnoses, understanding the relationship between cannabis use, varying doses, and anxiety is an important component for clinicians to understand before considering cannabis for management of endometriosis-related pain.^11,25^

The safety and side effects of cannabis use are important considerations for management of endometriosis pain. The safety profile of cannabis has been investigated by several studies, which have indicated relatively mild and manageable side effects, such as dizziness, dry mouth, and fatigue. However, it is essential to consider potential interactions with other medications and individual variations in response by each patient. Further research is needed to thoroughly evaluate the safety and long-term effects of cannabis use specifically in women with endometriosis. Despite how common endometriosis is, it’s hypothesized that the number of women with endometriosis is underreported as many women go undiagnosed for years owing to lack of education on endometriosis, diagnostic challenges, misdiagnosis due to overlap with other conditions, and gender biases.

## Conclusion

The current literature provides valuable insights into the potential use of cannabis for managing endometriosis-related pain. However, there are still gaps and limitations that remain necessary to address to enhance our understanding of the therapeutic potential and risks associated with cannabis use in women with endometriosis. Existing studies are limited by small sample sizes, variations in study design, and the predominance of preclinical and observational studies. Our findings emphasize the necessity of well-designed, blinded, randomized trials to ascertain the safety and efficacy of different cannabis dosages and administration methods that are legally available to determine the short- and long-term benefits and risks of using medical cannabis in the management of endometriosis.

## Abbreviations Used

CB: Cannabinoid Receptor
ECS: Endocannabinoid

## References

[1] Ellis K, Munro D, Clarke J. Endometriosis is undervalued: A call to action. Front Glob Womens Health 2022;3:902371; doi: 10.3389/fgwh.2022.902371 35620300 PMC9127440

[2] Nnoaham KE, Hummelshoj L, Webster P, et al. Impact of endometriosis on quality of life and work productivity: A multicenter study across ten countries. Fertil Steril 2011;96(2):366–373.e8; doi: 10.1016/j.fertnstert.2011.05.090 21718982 PMC3679489

[3] Vercellini P, Viganò P, Somigliana E, et al. Endometriosis: Pathogenesis and treatment. Nat Rev Endocrinol 2014;10(5):261–275; doi: 10.1038/nrendo.2013.255 24366116

[4] Hill KP. Medical marijuana for treatment of chronic pain and other medical and psychiatric problems: A clinical review. Jama 2015;313(24):2474–2483; doi: 10.1001/jama.2015.6199 26103031

[5] Glanville J, Foxlee R, Wisniewski S, et al. Translating the Cochrane EMBASE RCT filter from the Ovid interface to Embase.com: A case study. Health Info Libr J 2019;36(3):264–277; doi: 10.1111/hir.12269 31328866

[6] Glanville J, Dooley G, Wisniewski S, et al. Development of a search filter to identify reports of controlled clinical trials within CINAHL Plus. Health Info Libr J 2019;36(1):73–90; doi: 10.1111/hir.12251 30737884

[7] Lefebvre C, Glanville J, Briscoe S, et al., et al. Chapter 4: Searching for and selecting studies. In Higgins JPT, Thomas J, Chandler J(Eds), Cochrane Handbook for Systematic Reviews of Interventions version 6.3. Cochrane; 2022. Available from www.training.cochrane.org/handbook [Ovid, PubMed].

[8] Armour M, Sinclair J, Chalmers KJ, et al. Self-management strategies amongst Australian women with endometriosis: A National Online Survey. BMC Complement Altern Med 2019;19(1):17; doi: 10.1186/s12906-019-2431-x 30646891 PMC6332532

[9] Armour M, Sinclair J, Cheng J, et al. Endometriosis and cannabis consumption during the covid-19 pandemic: An International Cross-Sectional Survey. Cannabis Cannabinoid Res 2022;7(4):473–481; doi: 10.1089/can.2021.0162 35089093 PMC9418353

[10] Marcu I, Gee A, Lynn B. Cannabinoids and chronic pelvic pain in women: Focus on endometriosis. J Endometriosis Pelvic Pain Disord 2021;13(3):155–165; doi: 10.1177/22840265211011277

[11] Armour M, Sinclair J, Noller G, et al. Illicit cannabis usage as a management strategy in new zealand women with endometriosis: An online survey. J Womens Health (Larchmt) 2021;30(10):1485–1492; doi: 10.1089/jwh.2020.8668 33275491

[12] Sinclair J, Collett L, Abbott J, et al. Effects of cannabis ingestion on endometriosis-associated pelvic pain and related symptoms. PLoS One 2021;16(10):e0258940; doi: 10.1371/journal.pone.0258940 34699540 PMC8547625

[13] Sinclair J, Smith CA, Abbott J, et al. Cannabis use, a self-management strategy among australian women with endometriosis: Results from a National Online Survey. J Obstet Gynaecol Can 2020;42(3):256–261; doi: 10.1016/j.jogc.2019.08.033 31722852

[14] Allam S, Paris E, Lazcano I, et al. Detection of cannabinoid receptor expression by endometriotic lesions in women with endometriosis as an alternative to opioid-based pain medication. J Immunol Res 2022;2022:4323259; doi: 10.1155/2022/4323259 35692500 PMC9184153

[15] Liang AL, Gingher EL, Coleman JS. Medical cannabis for gynecologic pain conditions: A systematic review. Obstet Gynecol 2022;139(2):287–296; doi: 10.1097/AOG.0000000000004656 35104069

[16] Taylor C, Birch B. Cannabinoids in urology. Which benign conditions might they be appropriate to treat: A systematic review. Urology 2021;148:8–25; doi: 10.1016/j.urology.2020.10.024 33129871

[17] Tanaka K, Amoako AA, Mortlock S, et al. Gene expression of the endocannabinoid system in endometrium through menstrual cycle. Sci Rep 2022;12(1):9400; doi: 10.1038/s41598-022-13488-4 35672435 PMC9174470

[18] Leconte M, Nicco C, Ngô C, et al. Antiproliferative effects of cannabinoid agonists on deep infiltrating endometriosis. Am J Pathol 2010;177(6):2963–2970; doi: 10.2353/ajpath.2010.100375 21057002 PMC2993285

[19] Mlost J, Bryk M, Starowicz K. Cannabidiol for pain treatment: Focus on pharmacology and mechanism of action. Int J Mol Sci 2020;21(22):8870; doi: 10.3390/ijms21228870 33238607 PMC7700528

[20] Andrieu T, Chicca A, Pellegata D, et al. Association of endocannabinoids with pain in endometriosis. Pain 2022;163(1):193–203; doi: 10.1097/j.pain.0000000000002333 34001768 PMC8675052

[21] Sanchez AM, Quattrone F, Pannese M, et al. The cannabinoid receptor CB1 contributes to the development of ectopic lesions in a mouse model of endometriosis. Hum Reprod 2017;32(1):175–184; doi: 10.1093/humrep/dew281 27821707

[22] Bennici A, Mannucci C, Calapai F, et al. Safety of medical cannabis in neuropathic chronic pain management. Molecules 2021;26(20):6257; doi: 10.3390/molecules26206257 34684842 PMC8540828

[23] Mistry M, Simpson P, Morris E, et al. Cannabidiol for the management of endometriosis and chronic pelvic pain. J Minim Invasive Gynecol 2022;29(2):169–176; doi: 10.1016/j.jmig.2021.11.017 34839061

[24] Carrubba AR, Ebbert JO, Spaulding AC, et al. Use of cannabis for self-management of chronic pelvic pain. J Womens Health 2021;30(9):1344–1351; doi: 10.1089/jwh.2020.8737 33252316

[25] Petrie GN, Nastase AS, Aukema RJ, et al. Endocannabinoids, cannabinoids and the regulation of anxiety. Neuropharmacology 2021;195:108626; doi: 10.1016/j.neuropharm.2021.108626 34116110

